# Supplementary material for: Social groups buffer maternal loss in mountain gorillas
Source: eLife. 2021 Mar 23;10:e62939. doi: 10.7554/eLife.62939 (PMC7987338; doi:10.7554/eLife.62939)
Supplement: Supplementary file 1. [file elife-62939-supp1.docx]

**Supplementary Tables for:**

**Social groups buffer maternal loss in mountain gorillas**

Robin E Morrison^1,2^, Winnie Eckardt^1^, Fernando Colchero^3,4^, Veronica Vecellio^1^, Tara S Stoinski^1^

1. Dian Fossey Gorilla Fund, Rwanda
2. Centre for Research in Animal Behaviour, University of Exeter
3. Department of Mathematics and Computer Science, University of Southern Denmark
4. Interdisciplinary Center on Population Dynamics, University of Southern Denmark

**Supp. Table 1**. Cox-proportional hazards models showing the effects of the three maternal loss classes on survival. All results are relative to the non-orphan class.

|  | **Females** | |  |  | **Males** | |  |
| --- | --- | --- | --- | --- | --- | --- | --- |
| **Age-class** | Mean ± SE | P | |  | Mean ± SE | P | |
| Infants | 0.626 ± 0.585 | 0.284 | |  | -0.351 ± 0.549 | 0.523 | |
| Juveniles/subadults | 0.927 ± 1.140 | 0.415 | |  | 0.025 ± 1.050 | 0.981 | |

**Supp. Table 2.** The influence of age at maternal loss (infant or juvenile/subadult - J/SA) relative to non-orphans and dispersal prior to their first birth on a female’s age at first birth (n=53), modelled using a generalized linear model with a gaussian distribution.

|  | **Est ± SE** | **t** | **P** |
| --- | --- | --- | --- |
| **Intercept** | 1.495 ± 0.121 | 12.573 | <0.001 |
| **Dispersal** | -0.245 ± 0.197 | -1.239 | 0.222 |
| **Infant** | -0.154 ± 0.270 | -0.569 | 0.572 |
| **J/SA** | -0.552 ± 0.554 | -0.996 | 0.325 |
| **Dispersal:Infant** | 0.149 ± 0.382 | 0.390 | 0.698 |
| **Dispersal: J/SA** | 0.501 ± 0.611 | 0.820 | 0.416 |

**Supp. Table 3.** The influence of age at first birth, dispersal and age of maternal loss (I – infant, J/S –juvenile/sub-adult) on whether a female’s first offspring survived infancy (n=50), modelled using a binomial generalized linear model.

|  | **Est ± SE** | **Z** | **P** |
| --- | --- | --- | --- |
| **Intercept** | -1.402 ± 2.092 | -0.670 | 0.503 |
| **Age at birth** | 0.158 ± 0.198 | 0.799 | 0.424 |
| **Dispersal** | -0.264 ± 0.618 | -0.428 | 0.669 |
| **Maternal Loss I** | 0.397 ± 0.751 | 0.528 | 0.598 |
| **Maternal Loss J/S** | 0.375 ± 0.905 | 0.414 | 0.679 |

**Supp. Table 4.** Group size, composition and sampling during maternal loss periods. Network size and group composition represent the group members for which more than 12 focal scans were available in the 6 months both pre- and post-maternal loss (excluding the mothers of orphaned individuals). Where network size does not equal the sum of mature and immature gorilla columns this is due to infant gorillas under 2 years of age that are not examined within the orphan and non-orphan categories. Maternal loss incidents 1-19 were used to investigate whether orphans social network metrics change differently to non-orphans, as they included both orphaned and non-orphaned immature gorillas (aged 2-8 years, mean age ±SD of orphans: 5.12 ±1.49 years and non-orphans: 4.71 ±1.71 years). All maternal loss incidents (1-21) used to investigate how pairwise relationships change.

| **Maternal Loss Incident** | **Group** | **Date (d/m/y)** | **Network size** |  | **Immature gorillas** | |  | **Mature gorillas** | |  | **Focal Scans** | |
| --- | --- | --- | --- | --- | --- | --- | --- | --- | --- | --- | --- | --- |
|  |  |  |  |  | **Orphans** | **Non-orphans** |  | **Males** | **Females** |  | **Pre** | **Post** |
| 1 | BEE | 31/03/2007 | 22 |  | 1 | 6 |  | 6 | 8 |  | 2620 | 1820 |
| 2 | BWE | 27/07/2014 | 8 |  | 1 | 1 |  | 1 | 2 |  | 1704 | 1296 |
| 3 | ISA | 06/09/2012 | 10 |  | 1 | 2 |  | 2 | 5 |  | 2166 | 1967 |
| 4 | KUY | 12/02/2014 | 12 |  | 1 | 3 |  | 4 | 2 |  | 2274 | 720 |
| 5 | KUY | 25/06/2011 | 13 |  | 1 | 5 |  | 3 | 4 |  | 1545 | 1601 |
| 6 | KUY | 17/03/2013 | 13 |  | 1 | 3 |  | 4 | 3 |  | 1344 | 2421 |
| 7 | NTA | 07/06/2014 | 14 |  | 1 | 3 |  | 4 | 5 |  | 2466 | 2838 |
| 8 | NTA | 30/11/2015 | 8 |  | 1 | 2 |  | 3 | 2 |  | 912 | 1626 |
| 9 | PAB | 10/08/2013 | 31 |  | 3 | 10 |  | 9 | 9 |  | 1563 | 2297 |
| 10 | PAB | 03/03/2007 | 43 |  | 1 | 10 |  | 12 | 11 |  | 3311 | 3152 |
| 11 | PAB | 18/10/2006 | 54 |  | 1 | 11 |  | 14 | 19 |  | 4101 | 3820 |
| 12 | PAB | 18/11/2014 | 27 |  | 2 | 6 |  | 9 | 8 |  | 3072 | 3066 |
| 13 | PAB | 10/04/2007 | 43 |  | 2 | 10 |  | 12 | 11 |  | 3188 | 3158 |
| 14 | PAB | 30/12/2009 | 16 |  | 1 | 4 |  | 2 | 3 |  | 460 | 798 |
| 15 | PAB | 31/01/2007 | 48 |  | 4 | 11 |  | 12 | 16 |  | 3408 | 3648 |
| 16 | PAB | 16/02/2007 | 44 |  | 1 | 11 |  | 12 | 12 |  | 3425 | 3255 |
| 17 | SHI | 07/01/2008 | 25 |  | 1 | 5 |  | 7 | 10 |  | 2146 | 2806 |
| 18 | SHI | 14/06/2003 | 22 |  | 1 | 4 |  | 10 | 6 |  | 1513 | 1530 |
| 19 | UGE | 20/02/2013 | 7 |  | 3 | 1 |  | 2 | 1 |  | 1152 | 1768 |
| 20 | TIT | 06/09/2010 | 6 |  | 1 | 0 |  | 4 | 1 |  | 1167 | 876 |
| 21 | BWE | 07/10/2011 | 9 |  | 1 | 0 |  | 1 | 4 |  | 772 | 1193 |

**Supp. Table 5.** GAMMs predicting the change in network metric: a) eigenvector centrality, b) weighted degree and c) binary degree, for immature gorillas in social networks based on affiliative contact and proximity following an incident of maternal loss within the group.

Model: Metric Change ~ Orphan + Age + Deviance from group mean + s(focal scans), random=~(1|Network))

| a) Eigenvector centrality | | | |  |  | | |  |
| --- | --- | --- | --- | --- | --- | --- | --- | --- |
|  | **Affiliative Contact** | | |  | **Proximity** | | |  |
|  | Est ± SE | t | Pr(>\|t\|) | P_null_ | Est ± SE | t | Pr(>\|t\|) | P_null_ |
| Intercept | 0.406±0.080 | 5.069 | <0.001 |  | 0.058±0.057 | 1.019 | 0.310 |  |
| Orphan | -0.034±0.057 | -0.593 | 0.554 | 0.598 | 0.169±0.037 | 4.594 | <0.001 | <0.001 |
| Age | -0.029±0.014 | -2.111 | 0.037 |  | -0.006±0.009 | -0.655 | 0.514 |  |
| Deviance from group mean | -1.196±0.130 | -9.169 | <0.001 |  | -0.145±0.111 | -1.306 | 0.194 |  |
|  |  |  |  |  |  |  |  |  |
| **Smooth term** | edf | f | P |  | edf | F | P |  |
| s(focal scans) | 3.948 | 3.606 | 0.015 |  | 1.432 | 0.952 | 0.263 |  |
| b) Weighted degree (strength) | | | |  |  | | |  |
|  | **Affiliative Contact** | | |  | **Proximity** | | |  |
|  | Est ± SE | t | Pr(>\|t\|) | P_null_ | Est ± SE | t | Pr(>\|t\|) | P_null_ |
| Intercept | 0.115±0.075 | 1.541 | 0.126 |  | 0.114±0.051 | 2.261 | 0.025 |  |
| Orphan | 0.031±0.047 | 0.656 | 0.513 | 0.442 | 0.075±0.032 | 2.337 | 0.021 | 0.024 |
| Age | -0.024±0.011 | -2.092 | 0.038 |  | -0.016±0.008 | -2.024 | 0.045 |  |
| Deviance from group mean | -0.132±0.147 | -0.899 | 0.370 |  | -0.186±0.138 | -1.351 | 0.179 |  |
|  |  |  |  |  |  |  |  |  |
| **Smooth term** | edf | f | P |  | edf | F | P |  |
| s(focal scans) | 1 | 4.538 | 0.035 |  | 1.275 | 0.108 | 0.701 |  |
| c) Binary degree | | | |  |  | | |  |
|  | **Affiliative Contact** | | |  | **Proximity** | | |  |
|  | Est ± SE | t | Pr(>\|t\|) | P_null_ | Est ± SE | t | Pr(>\|t\|) | P_null_ |
| Intercept | 0.028±0.068 | 0.413 | 0.6805 |  | 0.016±0.031 | 0.507 | 0.613 |  |
| Orphan | 0.017±0.049 | 0.344 | 0.7312 | 0.438 | 0.042±0.024 | 1.766 | 0.080 | 0.086 |
| Age | -0.022±0.012 | -1.89 | 0.061 |  | -0.003±0.006 | -0.550 | 0.583 |  |
| Deviance from group mean | 0.366±0.180 | 2.04 | 0.0433 |  | 0.182±0.121 | 1.512 | 0.133 |  |
|  |  |  |  |  |  |  |  |  |
| **Smooth term** | edf | f | P |  | edf | F | P |  |
| s(focal scans) | NA* | | |  | 1 | 0.199 | 0.656 |  |

* Basis dimensions too small for inclusion of smoothing term

**Supp. Table 6.** GAMMs predicting the change in dyadic relationship strength (SRI values for affiliative contact and proximity) between all immature (I) gorillas including both orphans and non-orphans, and other group members (GM) following an incident of maternal loss within the group.

Model: Relationship Change ~ orphan_I * GM age sex class + age_I + sex_I + s(mean focal scans), random=~(1|Group/Network/ID_I)+(1|ID_GM))

|  | **Affiliative Contact** | | | **Proximity** | | |
| --- | --- | --- | --- | --- | --- | --- |
|  | Est ± SE | z | P | Est ± SE | z | P |
| Intercept | 0.006± 0.003 | 2.002 | 0.045 | 0.023 ± 0.014 | 1.635 | 0.102 |
| Orphan | 0.021 ± 0.003 | 6.660 | <0.001 | 0.029 ± 0.011 | 2.743 | 0.006 |
| Age of immature | -0.001 ± 0.000 | -3.544 | <0.001 | -0.002 ± 0.001 | -3.864 | <0.001 |
| Sex of immature (male) | 0.001 ± 0.001 | 1.025 | 0.306 | 0.006 ± 0.002 | 2.808 | 0.005 |
| *Group member age/sex class (relative to the dominant male)* | | | | | | |
| Adult male (subordinate) | -0.003 ± 0.002 | -1.491 | 0.136 | -0.017 ± 0.008 | -2.006 | 0.045 |
| Adult female | -0.002 ± 0.002 | -0.732 | 0.464 | -0.008 ± 0.009 | -0.936 | 0.349 |
| Blackback male | -0.001 ± 0.002 | -0.493 | 0.622 | -0.004 ± 0.009 | -0.418 | 0.676 |
| Subadult male | -0.002 ± 0.003 | -0.868 | 0.385 | 0.009 ± 0.010 | 0.864 | 0.388 |
| Subadult female | -0.001 ± 0.003 | -0.529 | 0.597 | -0.015 ± 0.011 | -1.399 | 0.162 |
| Juvenile | -0.001 ± 0.003 | -0.411 | 0.681 | -0.004 ± 0.010 | -0.394 | 0.693 |
| Infant | 0.003 ± 0.002 | 1.228 | 0.219 | 0.013 ± 0.009 | 1.377 | 0.169 |
| *Orphan and group member age/sex class interaction (relative to the dominant male)* | | | | | | |
| Adult male (subordinate) | -0.015 ± 0.004 | -4.238 | <0.001 | -0.014 ± 0.012 | -1.131 | 0.258 |
| Adult female | -0.021 ± 0.003 | -6.306 | <0.001 | -0.018 ± 0.011 | -1.599 | 0.110 |
| Blackback male | -0.023 ± 0.004 | -6.358 | <0.001 | -0.036 ± 0.012 | -2.972 | 0.003 |
| Subadult male | -0.025 ± 0.004 | -6.200 | <0.001 | -0.033 ± 0.014 | -2.418 | 0.016 |
| Subadult female | -0.015 ± 0.004 | -3.812 | <0.001 | 0.009 ± 0.014 | 0.681 | 0.496 |
| Juvenile | -0.021 ± 0.004 | -5.386 | <0.001 | 0.002 ± 0.013 | 0.148 | 0.882 |
| Infant | -0.025 ± 0.004 | -7.032 | <0.001 | -0.024 ± 0.012 | -2.030 | 0.042 |

Smooth term: s(mean focal scans) Contact: F=3.866, p=0.049 Proximity: F=3.006, p=0.034

**Supp. Table. 7.** GAMMs predicting the change in dyadic relationship strength (SRI values for affiliative contact and proximity) between adult males (AM) and orphans (O) following maternal loss.

Model: Relationship Change ~ Age_O + Sex_O + Father + Sibling * Dominant_AM + s(MeanDen), random=~(1|ID_O) + (1|ID_AM)

|  | Affiliative Contact | | | Proximity | | |
| --- | --- | --- | --- | --- | --- | --- |
|  | Est ± SE | Z | P | Est ± SE | z | P |
| Intercept | 0.008 ± 0.009 | 0.881 | 0.381 | 0.017 ± 0.029 | 0.604 | 0.548 |
| *Orphan* | | | | | | |
| Orphan age | -0.002 ± 0.001 | -1.051 | 0.296 | -0.003 ± 0.005 | -0.643 | 0.522 |
| Orphan Sex (male) | 0.002 ± 0.004 | 0.550 | 0.584 | 0.001 ± 0.013 | 0.048 | 0.962 |
| *Adult Male* | | | | | | |
| Father | 0.009 ± 0.008 | 1.130 | 0.262 | -0.014 ± 0.026 | -0.552 | 0.583 |
| Maternal sibling | 0.027 ± 0.007 | 3.807 | <0.001 | 0.051 ± 0.023 | 2.237 | 0.028 |
| Dominant male | 0.008 ± 0.009 | 0.859 | 0.393 | 0.084± 0.030 | 2.768 | 0.007 |
| *Dominance-kin interaction* | | | | | | |
| Dominant sibling | -0.039 ± 0.013 | -2.932 | 0.004 | -0.074 ± 0.041 | -1.765 | 0.082 |

Smooth term: s(mean focal scans) Contact: F=4.751, p=0.032 Proximity: F=3.023, p=0.087
